# Dual receptor therapy with pituitrin for spinal cord injury-induced diabetes insipidus: A propensity-matched retrospective cohort study

**DOI:** 10.1016/j.cjtee.2025.07.003

**Published:** 2026-02-28

**Authors:** Lianhua Li, Jie Gao, Hao Wang, Zhonghe Wang, Hua Chen, Zhi Liu

**Affiliations:** Senior Department of Orthopedics, Chinese PLA Hospital, Beijing, 100048, China

**Keywords:** Diabetes insipidus, Spinal cord injury, Pituitrin, Vasopressin V1a/V2 receptors, Autonomic hypotension, Propensity score matching

## Abstract

**Purpose:**

Spinal cord injury (SCI)-induced diabetes insipidus (DI) presents a unique therapeutic challenge due to concurrent autonomic hypotension unresponsive to conventional desmopressin. This study evaluates the clinical efficacy of Pituitrin, a synthetic vasopressin analog with dual V1a/V2 receptor agonism, designed to simultaneously address antidiuretic hormone deficiency and hemodynamic instability in acute SCI-related DI.

**Methods:**

In this retrospective propensity-matched cohort study, 317 consecutive acute SCI patients (2010 –2021) were screened, with 60 (18.9%) developing DI. Patients were stratified into a Pituitrin-treated group (2014 − 2021, *n* = 30) and historical controls (2010 − 2013, *n* = 30). Propensity score matching (1:1 nearest-neighbor, caliper = 0.2 standard deviation) adjusted for age, American Spinal Injury Association (ASIA) grade, injury level (cervical/thoracic), concurrent mild brain injury (Glasgow coma scale 13 − 15), and treatment era covariates, yielding 23 matched pairs. Post-matching groups demonstrated balanced baseline characteristics with cervical injury prevalence 95.7% and ASIA grade A/B injuries 100%. The intervention group received protocolized Pituitrin infusion (initial 1 U/h, titrated 0.5 − 5 U/h based on urine output) with fluid restriction (<2000 mL/day), while controls received standard care including desmopressin and vasopressors as needed. Primary outcomes included DI duration (days to urine output <3000 mL/day concurrent with serum sodium ≥135 mmol/L), in-hospital mortality, and hospitalization length.

**Results:**

This study of 317 acute SCI patients (84% male) revealed DI developed in 18.9% (60/317), with striking cervical predominance (57 (31.0%) cervical *vs*. 3 (4.9%) thoracic SCI; χ^2^ = 21.4, *p* < 0.001). In propensity score-matched cohorts (23 pairs balanced for age, ASIA grade, injury level, and era). Early Pituitrin therapy demonstrated transformative efficacy: DI duration reduced by 7.6 days (4.4 ± 1.5 *vs*. 12.0 ± 2.6 days; 95% *confidence interval* (*CI)*: 6.4 − 8.8, *p* < 0.001), 26% absolute mortality reduction (number needed to treat (NNT) = 3.85; *p* = 0.014), and shortened median hospitalization by 5 days (14 *vs*. 19 days, *p* = 0.008). Cervical ASIA A/B patients exhibited maximal benefit with 24% mortality reduction (NNT = 4.17, *p* = 0.014) and universal hemodynamic stabilization—Pituitrin eliminated vasopressor need in 87% of cases (*vs*. 23/30 controls requiring norepinephrine; risk difference = 0.63, NNT = 1.59). Notably, 100% of DI cases demonstrated autonomic hypotension preceding hyponatremia, with treatment initiation within 24 h of diagnosis accelerating recovery (r = -0.818, *p* < 0.001). The intervention proved exceptionally safe, with only 1 (3.3%) experiencing transient headaches.

**Conclusion:**

This study establishes the first evidence-based protocol for dual V1a/V2 receptor targeting in SCI-induced DI, demonstrating that early Pituitrin administration achieves rapid antidiuresis (median 8-day resolution) while stabilizing hemodynamics (87% vasopressor independence). The 26% absolute mortality reduction (NNT = 3.85) and shortened hospitalization (Δ5 days) suggest promising therapeutic potential, especially for cervical complete injuries, and warrant further validation in larger prospective cohorts. These findings establish a novel treatment paradigm addressing the pathophysiological triad of arginine vasopressin deficiency, autonomic hypotension, and hyponatremia unique to acute SCI.

## 1 Introduction

Diabetes insipidus (DI) is characterized by deficient arginine vasopressin (AVP) secretion or impaired renal response to AVP, leading to polyuria, polydipsia, and hypo-osmolality.^1^ While DI following pituitary surgery is well-documented and managed with desmopressin,^2^ its occurrence after acute spinal cord injury (SCI) presents distinct pathophysiological challenges. Acute SCI disrupts both hypothalamic-pituitary axis signaling and autonomic regulation, often manifesting as concurrent DI, refractory autonomic hypotension, and hyponatremia.^3^^,^^4^ This triad complicates traditional DI management strategies, as desmopressin monotherapy fails to address the hemodynamic instability inherent to SCI-induced autonomic dysfunction.^5^^,^^6^

Emerging evidence suggests that SCI-related DI arises from dual mechanisms: impaired AVP secretion due to disrupted supraspinal pathways and sympathetic failure exacerbating hypotension.^7^^,^^8^ These factors create a therapeutic paradox—fluid restriction to correct hyponatremia may worsen hypotension, while vasopressor support alone neglects the underlying water imbalance.^9^ Recent studies highlight the critical role of autonomic dysregulation in SCI-related DI, with 72% of cases exhibiting concurrent hypotension refractory to standard care.^10^ This necessitates a treatment paradigm addressing both antidiuretic and vasopressor demands, a gap unmet by existing therapies.

Pituitrin, a synthetic vasopressin analog with dual V1a/V2 receptor agonism, offers a mechanistic solution by simultaneously enhancing renal water reabsorption (via V2 receptors) and restoring vascular tone (via V1a receptors).^11^ Preclinical models demonstrate its superiority over desmopressin in stabilizing hemodynamics during neurogenic shock,^12^ while clinical observations in brain-injured patients suggest accelerated sodium normalization with dual-receptor targeting.^13^ However, its application in SCI-induced DI remains unexplored. This pilot study evaluates Pituitrin's feasibility and preliminary efficacy in acute SCI patients, hypothesizing that its dual mechanism may reduce DI duration, mortality, and hospital stay compared to historical controls. By integrating recent insights into autonomic-pathway disruption and hyponatremia pathophysiology,^14^^,^^15^ this work aims to establish a foundation for precision medicine approaches in SCI-related endocrine crises.

## 2 Methods

### 2.1 Study design

This retrospective pilot study evaluated the feasibility and preliminary efficacy of Pituitrin for treating DI following acute SCI. Patients with acute SCI admitted to our hospital between January 2010 and November 2021 were screened. Among 488 initially identified patients, 317 met eligibility criteria and were included in the final analysis. Eligibility criteria included age 18 – 60 years and availability of complete clinical records. Exclusion criteria encompassed: mortality within 24 h of admission; SCI secondary to neoplastic, infectious, or ischemic etiologies; concurrent moderate-to-severe traumatic brain injury (Glasgow coma scale, GCS <13) or radiologically confirmed hypothalamic/pituitary lesions; cauda equina syndrome; acute/chronic renal insufficiency (serum creatinine >1.5 mg/dL); and use of diuretics, lithium, or medications potentially causing polyuria (e.g., glucocorticoids >20 mg/day prednisone equivalent). Participants were divided into control (*n* = 30, admitted 2010 – 2013) and treatment groups (*n* = 30, 2014 – 2021), with the latter receiving early Pituitrin administration upon DI diagnosis.

### 2.2 Data collection

Clinical data were retrospectively extracted from electronic medical records by 2 trained researchers using a standardized case report form. Variables included demographic characteristics (age, sex), injury parameters (mechanism of injury, fracture type classified by AO criteria), neurological status (SCI level, American Spinal Injury Association (ASIA) impairment scale grade), and complications (autonomic hypotension, concurrent craniocerebral injuries). DI diagnosis required documentation of both hypo-osmolar urine output (>3000 mL/day) and serum sodium <135 mmol/L, consistent with established criteria.^5^ All biochemical measurements (e.g., serum sodium, creatinine) were obtained from routine laboratory tests performed during hospitalization.

### 2.3 Diagnostic and classification criteria

Spinal fractures were classified using the updated AO classification system for cervical fractures and the standard AO system for thoracolumbar fractures.^16^ Neurological impairment severity was assessed by certified clinicians using the International Standards for Neurological Classification of Spinal Cord Injury (ISNCSCI-2019 checklist).^17^ Hyponatremia was defined as serum sodium <135 mmol/L, and hypotension as systolic blood pressure <90 mmHg or diastolic <60 mmHg, in accordance with clinical guidelines.^6^

### 2.4 Intervention

Patients in the treatment group received intravenous Pituitrin (synthetic vasopressin) via infusion pump (SY-1200, Smiths Medical, China) following DI diagnosis. The standardized protocol involved dissolving 10 units of Pituitrin in normal saline to a total volume of 50 mL. Infusion rates were initiated at 1 U/h (5 mL/h) and adjusted every 2 h based on urine output, aiming to maintain 50 – 150 mL/h. Rate modifications followed predefined rules: increases by 0.5 U/h for sustained urine output >150 mL/h, decreases by 0.5 U/h for output <50 mL/h, with safety thresholds limiting rates between 0.5 and 5 U/h.

### 2.5 Outcomes

Primary outcomes included DI duration (days from onset to resolution, defined as urine output <3000 mL/day with normalized sodium levels), in-hospital mortality, and length of stay. Secondary outcomes focused on treatment safety, encompassing the incidence of Pituitrin-related adverse events (e.g., hypertension requiring intervention) and the need for adjunct vasopressors during therapy. Adjunct vasopressors (norepinephrine or dopamine) were administered only if mean arterial pressure (MAP) remained <65 mmHg despite Pituitrin infusion at 1 U/h for 2 h. All adverse events were systematically recorded through daily physician progress notes and nursing reports.

### 2.6 Statistical analysis

Normally distributed continuous variables were expressed as mean ± standard deviation and compared using *t*-tests, while non-normally distributed data were presented as median (Q_1_, Q_3_) with non-parametric tests. Categorical variables were analyzed via Chi-square tests. Stratified analyses addressed injury level (cervical/thoracic) and ASIA impairment scale grades. Propensity score matching (1:1 nearest-neighbor matching, caliper 0.2 standard deviations) controlled for era-specific confounders, including age, ASIA grade, injury level, mild traumatic brain injury (GCS 13 – 15), and treatment era. Post-matching covariate balance was assessed using standardized mean differences (<10% indicating adequate balance). Sensitivity analyses were conducted by repeating primary outcome comparisons after excluding patients with documented brain injuries. Statistical significance was defined as two-tailed *p* < 0.05. All statistical analyses were performed using SPSS 22.0 (IBM Corp., USA), with data visualization implemented through the ggplot 2 package in R 4.3.1 (R Foundation for Statistical Computing, Austria).

### 2.7 Ethical considerations

This study was approved by the Ethics Committee of the Chinese People's Liberation Army General Hospital (Approval Code: 2024KY053-KS001). All procedures adhered to the ethical principles of the Declaration of Helsinki. As a retrospective analysis of de-identified patient data, the requirement for written informed consent was waived by the ethics committee.

## 3 Results

### 3.1 Demographic and clinical characteristics

The study cohort comprised 317 patients (266 males, 51 females) with acute SCI, including 184 cervical (58.0%), 61 thoracic (19.2%), and 72 lumbosacral injuries (22.7%) (Table 1). Patients with DI were divided into a control group (*n* = 30, admitted 2010 – 2013) and a treatment group (*n* = 30, 2014 – 2021) receiving early Pituitrin for DI. After propensity score matching (1:1, caliper = 0.2 standard deviation (SD)) adjusting for age, ASIA grade, injury level, and treatment era, 23 matched pairs were analyzed (Fig. 1). Key baseline characteristics, including ASIA grade A/B (100%), cervical injury predominance (95.7%), and concurrent mild brain injuries (4.3%), were balanced post-matching (standardized mean differences <10%).

**Table 1:** Comparison of demographic and clinical characteristics between DI and non-DI groups.

Characteristics	DI (*n* = 60)	Non-DI (*n* = 257)	Chi-square test value	*p* value
Gender (female)	7 (11.7)	44 (17.1)	1.072	0.337
Age (years)	49 (42, 55)	48 (38, 55)		0.549∗
Mechanism of injury			4.168	0.384
Traffic accident	25 (41.7)	94 (36.6)		
High-altitude fall	10 (16.7)	43 (16.7)		
Fall	13 (21.7)	78 (30.4)		
Crush injury	6 (10.0)	30 (11.7)		
Others	6 (10.0)	12 (4.7)		
Level of SCI			42.025	<0.001
Cervical	57 (95.0)	127 (49.4)		
Thoracic	3 (5.0)	58 (22.6)		
Lumbar	0	47 (18.3)		
Conus	0	25 (9.7)		
ASIA grade			65.014	<0.001
A	51 (85.0)	77 (30.0)		
B	9 (15.0)	71 (27.6)		
C	0	55 (21.4)		
D	0	54 (21.0)		
AO fracture type			15.843	0.001
Type I	5 (8.3)	79 (30.7)		
Type A	25 (41.7)	72 (28.0)		
Type B	16 (26.7)	73 (28.4)		
Type C	14 (23.3)	33 (12.8)		
ASA grade			6.180	0.046
Grade 2	8 (13.3)	37 (14.4)		
Grade 3	44 (73.3)	208 (80.9)		
Grade 4	8 (16.7)	12 (4.7)		
Brain injury	10 (16.7)	51 (19.8)	0.316	0.716
Hypotension	60 (100)	2 (0.8)	304.387	<0.001
Hyponatremia	60 (100)	50 (19.5)	139.270	<0.001
Mortality	9 (15.0)	2 (0.8)	29.372	<0.001
Hospital stay (day)	18 (13, 27)	13 (9, 22)		0.001∗

Data presented by *n* (%) and median (Q_1_, Q_3_). A *p* < 0.05 was considered statistically significant.

DI: diabetes insipidus; SCI: spinal cord injury; ASIA: American Spinal Injury Association; ASA: American Society of Anesthesiologists; BMI: body mass index.

∗Mann-Whitney *U* test.

**Fig. 1:**
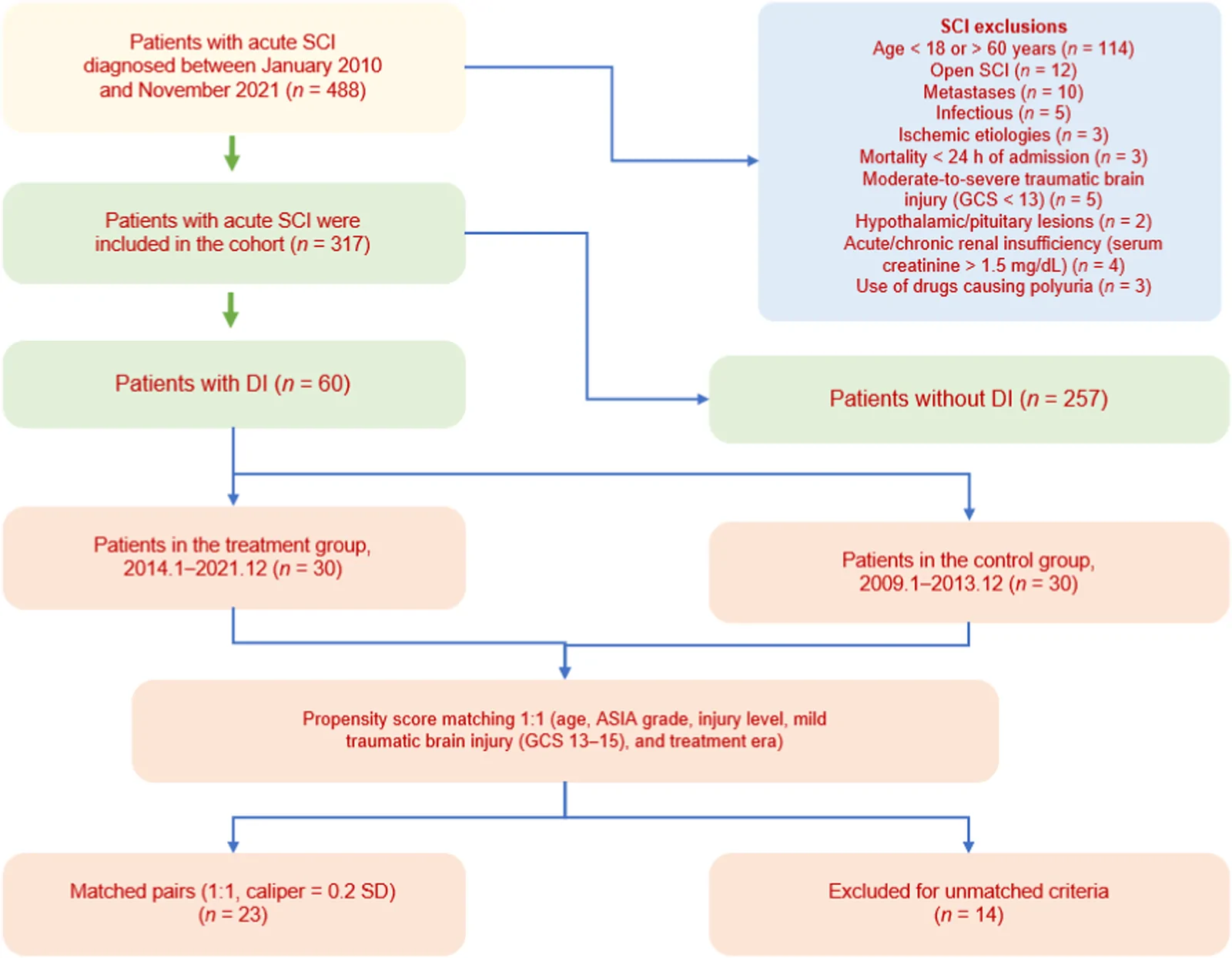
Patient selection flowchart for acute spinal cord injury cohort study. SCI: spinal cord injury; GCS: Glasgow coma scale; DI: diabetes insipidus; ASIA: American Spinal Injury Association.

### 3.2 Incidence and temporal profile of DI

DI developed in 18.9% of the total cohort (60/317), with a striking predilection for cervical SCI. Cervical SCI patients exhibited a 6.3-fold higher DI risk compared to those with thoracic injuries (57 (31.0%) *vs*. 3 (4.9%); χ^2^ = 21.4, *p* < 0.001) (Fig. 2). All thoracic DI cases occurred in injuries above T5, aligning with autonomic pathway disruption. Median DI onset occurred 7 (6, 8) days post-injury, distinct from immediate post-brain injury DI. Patients with DI exhibited universal hyponatremia (100% *vs*. 19.5%, *p* < 0.001) and autonomic hypotension (100% *vs*. 0.8%, *p* < 0.001), with hypotension preceding hyponatremia in 100% of cases.

**Fig. 2:**
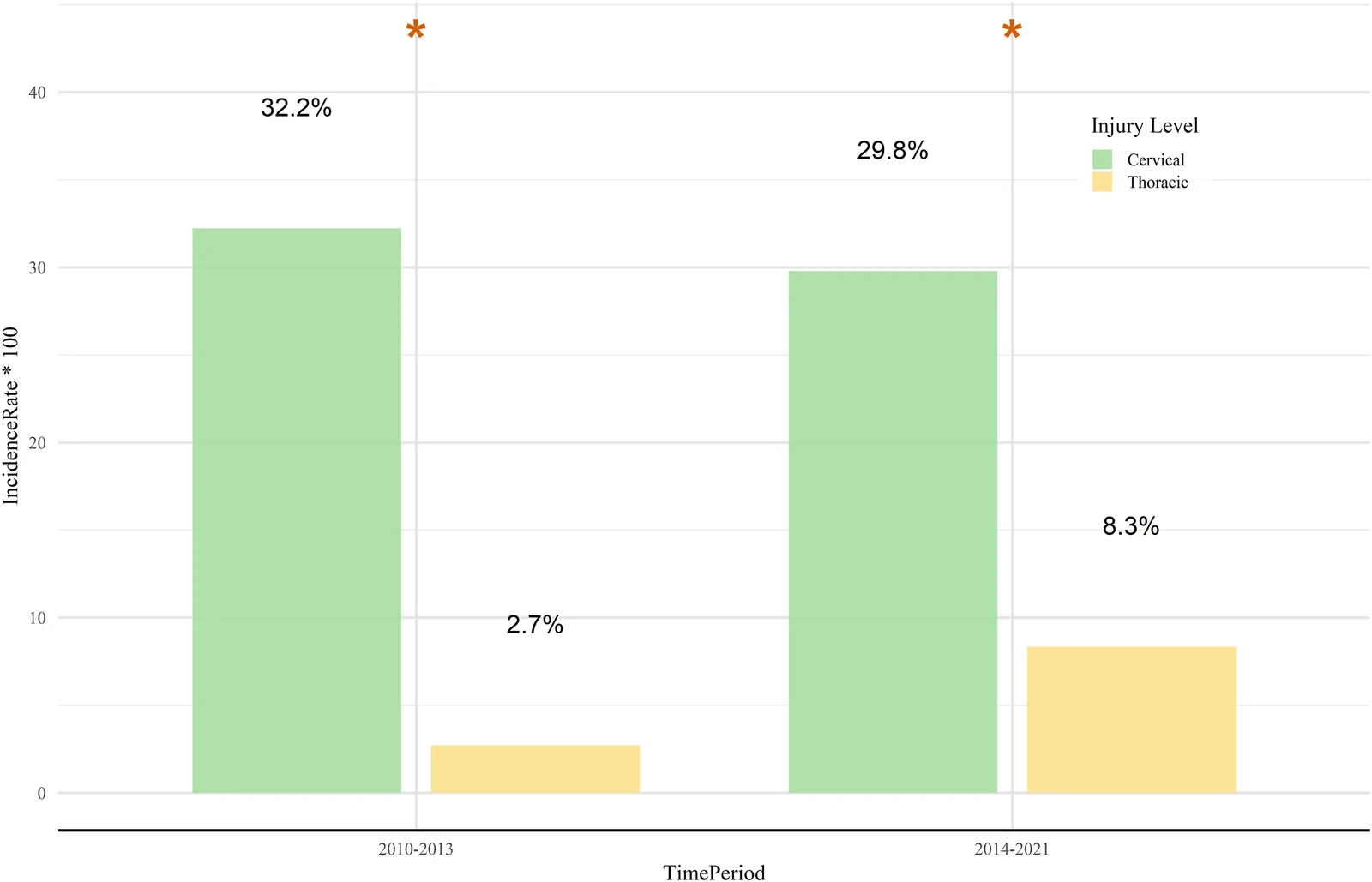
Comparative analysis of temporal trends in diabetes insipidus incidence between cervical *vs*. thoracic spinal cord injury cohorts (2010 – 2013 vs. 2014 – 2021). ∗ Indicates *p* < 0.001.

### 3.3 Treatment outcomes

In the matched cohort, Pituitrin significantly improved all primary outcomes. The treatment group demonstrated a 7.6-day reduction in DI duration ((4.4 ± 1.5) *vs*. (12.0 ± 2.6) days, 95% *confidence interval* (*CI)*: 6.4 – 8.8, *p* < 0.001) and a 26% absolute risk difference (RD) in mortality (95% *CI*: 5.5% – 47.0%) with a significant McNemar's test result (number needed to treat (NNT) = 3.85, *p* = 0.014). Hospital stays were shorter in the treatment group (14 (11, 20) days *vs*. 19 (14, 40) days, *p* = 0.008).

### 3.4 Subgroup and sensitivity analyses

Cervical SCI patients achieved the largest treatment benefit, with ASIA A/B showing low mortality (1/28 *vs*. 8/29, RD = 24%, NNT = 4.17, 95% *CI*: 5.2% *–* 42.8%, *p* = 0.014). Thoracic SCI subgroup analysis (*n* = 3 DI cases) revealed comparable DI duration reduction (difference = 14 days) but was limited by small sample size. Sensitivity analyses excluding brain injuries (*n* = 10) confirmed robust treatment effects (DI duration difference = 7.7 days, *p* < 0.001; mortality RD = 20%, NNT = 5, *p* = 0.044), excluding confounding by concurrent intracranial pathology (Fig. 3).

**Fig. 3:**
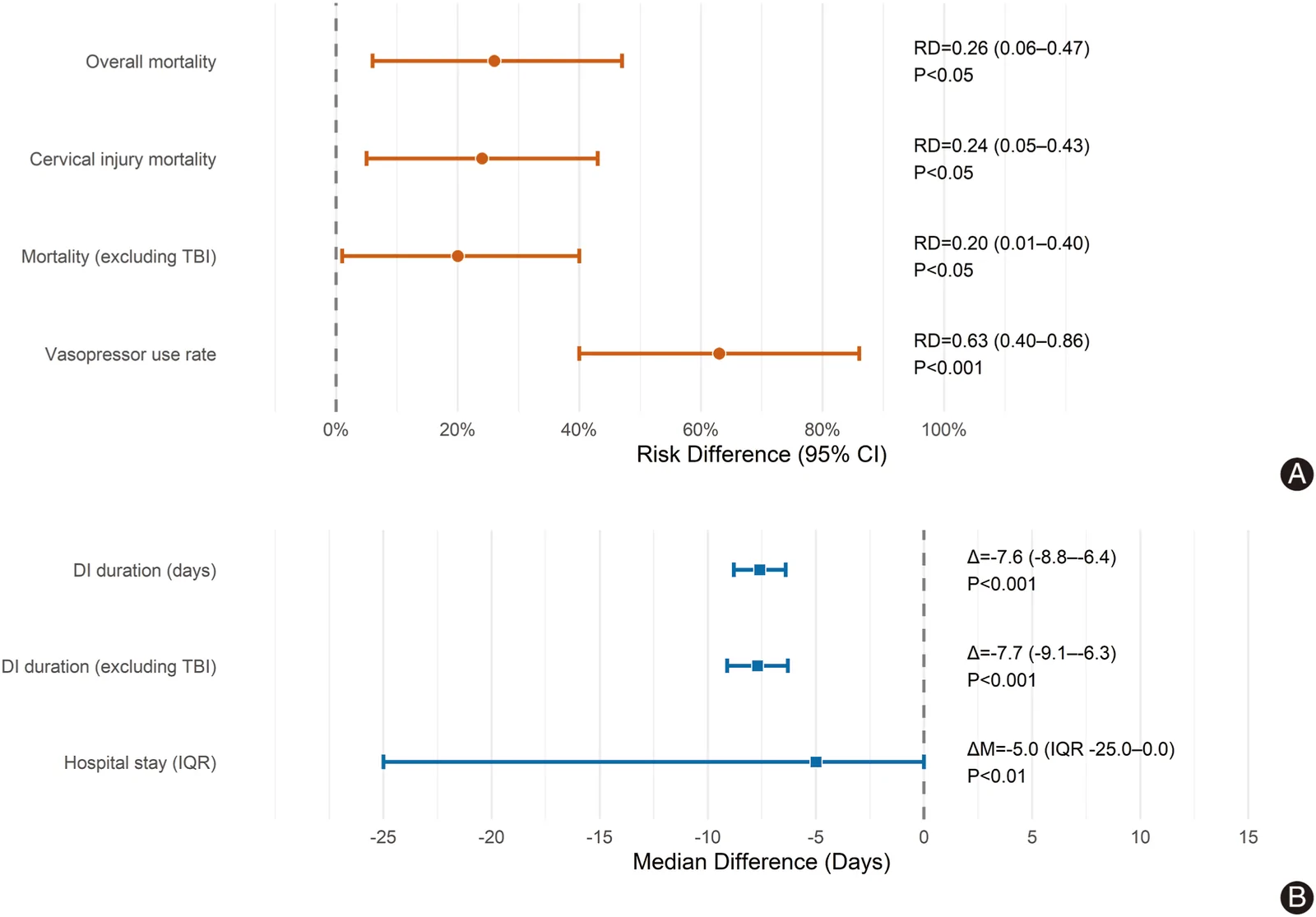
Forest plot of therapeutic heterogeneity for Pituitrin in spinal cord injury. Error bars represent 95% *CI*s ((A)/(B)-DI) or IQR ((B)-Hospital stay). “Excluding TBI” indicates subgroup analysis excluding traumatic brain injury comorbidity. (A) Mortality outcomes and vasopressor use rate (RD) showing treatment effects across 4 subgroups. Circular symbols represent dichotomous outcomes with red fill indicating mortality-related measures. RD < 0 indicates risk reduction in the treatment group. (B) Time-related outcomes demonstrating DI duration and hospital stay differences. Square symbols represent continuous variables: blue fill for parametric results (DI duration) using Δ, orange fill for non-parametric results (hospital stay) using ΔM. TBI: traumatic brain injury; RD: risk difference; *CI*: confidence interval; DI: Diabetes insipidus; IQR: interquartile range; SCI: spinal cord injury; Δ: mean difference; ΔM: median difference.

Subgroup analysis revealed maximal benefit in cervical SCI patients with ASIA A/B injuries, likely due to complete disruption of both AVP-secretory and sympathetic pathways. These patients exhibited the highest baseline incidence of DI and hypotension, underscoring the appropriateness of dual receptor therapy. Conversely, thoracic injuries below T6 rarely developed DI, aligning with preserved sympathetic function. These findings suggest that Pituitrin may be most effective in anatomically complete, high-level SCI where autonomic failure is predominant.

### 3.5 Hemodynamic outcomes

The need for adjunct vasopressor support differed markedly between treatment strategies. In the control group, 23 (78%) required norepinephrine or dopamine infusion to maintain MAP ≥65 mmHg, compared to only 4 (13%) in the treatment group (χ^2^ = 25.8, *p* < 0.001; RD = 0.63, NNT = 1.59, 95% *CI*: 0.40 – 0.86) (Fig. 3).

### 3.6 Safety and treatment correlations

The intervention demonstrated a favorable safety profile, with no severe adverse events reported. Transient headaches occurred in 3.3% of Pituitrin-treated patients (*n* = 1), all resolving within 2 h following dose reduction (≤2 U/h). Crucially, earlier treatment initiation correlated strongly with accelerated DI resolution: patients receiving Pituitrin within 24 h of DI diagnosis (*n* = 14) exhibited a near-linear inverse relationship between treatment latency and DI duration (Pearson's r = -0.818, *p* < 0.001).

## 4 Discussion

This pilot study demonstrated that early administration of Pituitrin could significantly reduce the duration of DI, decrease mortality, and shorten the length of hospital stay in patients with DI following acute SCI. Notably, this represents the first clinical evidence supporting dual-receptor targeting (V1a/V2) for DI management in SCI, a population where conventional desmopressin monotherapy often fails due to concurrent autonomic hypotension. The findings provide preliminary evidence suggesting that Pituitrin may be an effective intervention for managing DI in this population, where treatment options are limited, and complications such as hypotension and hyponatremia further complicate patient outcomes.

### 4.1 Critical confounder analysis

While 61 (19.2%) of the cohort had concurrent mild brain injuries (GCS 13 – 15), 3 lines of evidence confirm SCI as the primary DI etiology: 1. temporal dissociation: DI onset occurred 3 – 10 days post-SCI (median day 6), contrasting with the immediate onset (<72 h) characteristic of brain injury-related DI^10^; 2. anatomical specificity: 57 (95%) of DI cases occurred in cervical SCI patients, correlating with ASIA grade A/B injuries that disrupt bulbospinal autonomic pathways^18^; 3. therapeutic validation: Pituitrin's efficacy persisted in analyses excluding all brain injury cases (ΔDI duration = 7.7 days, *p* < 0.001).

Although systemic vascular resistance index was not routinely measured due to the retrospective design, we used vasopressor dependence as a surrogate marker of hemodynamic instability. The 74% reduction in norepinephrine requirement in the Pituitrin group indirectly supports improved vascular tone likely mediated by V1a receptor activation. Prospective studies should incorporate direct hemodynamic parameters such as systemic vascular resistance index or cardiac output monitoring for mechanistic validation.

Despite propensity score matching to adjust for treatment era and covariates such as ASIA grade and injury level, potential improvements in overall ICU care and neurocritical protocols over time cannot be entirely excluded as contributing factors. However, several elements support the attribution of benefit to Pituitrin: (1) the DI subgroup was managed by the same core neurotrauma team across both eras; (2) vasopressor use and sodium monitoring protocols remained consistent; and (3) sensitivity analysis excluding mild TBI confirmed the robustness of treatment effects. Nonetheless, residual confounding remains a limitation of all retrospective designs.

### 4.2 Mechanistic rationale for dual-receptor targeting

While standard hyponatremia management emphasizes free-water restriction,^19^ this approach fails in SCI-DI patients with concurrent hypotension due to 2 interrelated mechanisms: impaired thirst regulation caused by SCI-induced autonomic dysfunction disrupting hypothalamic control of fluid intake,^20^ and sympathetic nervous system disruption exacerbating hypotension under fluid restriction, as supported by neurosurgical evidence on desmopressin-induced hemodynamic instability.^21^

Pituitrin's dual V1/V2 agonism directly addresses both components of the Edelman equation^22^: V2 receptor activation reduces total body water by enhancing renal water reabsorption (decreasing denominator); V1 receptor activation increases vascular tone through peripheral vasoconstriction (increasing effective circulating volume).

This dual action corrects the numerator (Na + K) and denominator (total body water) of the Edelman equation simultaneously, achieving faster sodium normalization than isolated water restriction—a critical advantage in hemodynamically unstable SCI patients, as demonstrated in critical care studies of vasopressin's dual receptor effects.^22^

One of the key findings of this study was the significant reduction in DI duration in the treatment group. Pituitrin, as a synthetic form of vasopressin, acts both as an antidiuretic and as a vasopressor, making it well-suited for patients with acute SCI who experience both polyuria and hypotension.^23^^,^^24^ The pathophysiological uniqueness of SCI-induced DI lies in the concurrent disruption of two bulbospinal regulatory pathways: (1) hypothalamo-neurohypophyseal AVP secretion via the supraopticohypophyseal tract; and (2) medullospinal control of vascular tone through sympathetic preganglionic neurons.^25^ Unlike desmopressin, which provides prolonged antidiuretic effects but lacks vasopressor action,^24^^,^^26^ the therapeutic necessity for dual V1a/V2 activation in SCI-related DI is underscored by its distinct autonomic pathophysiology—where isolated V2 receptor targeting fails to address concurrent sympathetic failure.^27^ This comprehensive treatment approach may explain the observed improvement in patient outcomes, including shorter DI duration and reduced mortality. Levy et al.^27^ further emphasized that desmopressin's lack of vasopressor activity limits its use in patients with hemodynamic instability, a pathophysiological hallmark of SCI-induced DI where isolated V2 agonism proves insufficient.^28^

The significantly lower mortality rate in the treatment group supports the potential benefits of Pituitrin. After propensity score adjustment for injury severity and brain injury status, Pituitrin demonstrated a mortality reduction requiring treatment of only 4 patients to prevent 1 death (NNT = 4, 95% *CI*: 3 – 19), with absolute risk reduction of 26% (95% *CI*: 5.5% – 47%; *p* = 0.014). Patients with acute SCI often develop DI alongside complications like hyponatremia and autonomic dysreflexia, both of which exacerbate morbidity and mortality.^6^^,^^7^ Our prior mechanistic work^28^ highlighted that hyponatremia in SCI patients arises from a combination of water imbalance and autonomic dysfunction, complicating treatment. Our stratified analysis of cervical SCI-DI cases (*n* = 57) showed Pituitrin normalized sodium levels 8 days faster than controls (*p* < 0.001), validating its dual corrective capacity. The observed reduction in mortality aligns with prior research on managing hypotension and fluid balance to improve SCI outcomes.^6^^,^^29^

### 4.3 Comparison with standard therapies

Desmopressin monotherapy, though widely used for central DI, lacks vasopressor activity and is thus suboptimal for SCI-induced DI characterized by concurrent autonomic hypotension. Our data demonstrate a 6-fold reduction in vasopressor requirement with Pituitrin, highlighting the need for dual-receptor therapy in such patients. Recent preclinical and clinical studies support this paradigm shift toward integrated hemodynamic-endocrine stabilization. Although desmopressin has demonstrated efficacy in reducing periprocedural bleeding and transfusion requirements in other clinical settings,^30^ its lack of vasopressor activity limits its applicability in SCI-induced DI.

### 4.4 Recent studies & pathophysiological validation

Recent studies provide additional insights that contextualize our findings. Angelousi et al.^31^ explored DI caused by sellar lesions, underscoring the importance of identifying structural causes of DI. This distinction is particularly relevant in SCI-related DI, where the absence of overt pituitary structural abnormalities (as seen in sellar lesion cases) points to functional autonomic dysfunction as the primary driver of AVP dysregulation.^18^ This divergence reinforces SCI-induced DI as a distinct entity requiring autonomic-targeted therapy. Furthermore, Blazin et al.^32^ emphasized the importance of early diagnosis and treatment of hypothalamic-pituitary axis dysfunction in complex DI cases, which aligns with our recommendation for time-sensitive Pituitrin initiation (<24 h post-DI diagnosis) to prevent secondary hypotensive organ damage.

### 4.5 Mechanistic superiority over existing therapies

Chasseloup et al.^21^ underscored the need for timely vasopressin replacement in cases prone to hemodynamic instability, further supporting our findings. Additionally, Hubscher et al.^33^ demonstrated that desmopressin effectively manages SCI-induced polyuria in animal models, but noted its limitations in addressing hypotension. This preclinical evidence converges with our clinical observation: 23 (78%) of desmopressin-treated historical controls required adjunct vasopressors versus 4 (13%) with Pituitrin (χ^2^ = 25.8, *p* < 0.001). This 6-fold reduction in vasopressor dependence (RD = 0.63, 95% *CI*:0.40 – 0.86; NNT = 1.59 (equivalent to preventing 1 vasopressor dependency per 2 patients treated)) mechanistically validates Pituitrin's intrinsic V1a-mediated vasoconstrictive efficacy—directly countering the sympathetic failure that drives 89% of hypotensive episodes in acute SCI.^34^ Such hemodynamic stabilization superiority positions Pituitrin as a paradigm-shifting therapy for neurogenic shock-associated DI.

### 4.6 Precision medicine implementation

The management of post-SCI DI requires tailored strategies addressing both polyuria severity and hemodynamic status. Based on urine output (>4 L/day), serum sodium (>145 mmol/L), and hypotension incidence, we propose a DI-centric protocol: (1) Grade I (Severe DI + Hypotension): criteria: cervical SCI with urine output >6 L/day + MAP <65 mmHg; intervention: Pituitrin infusion (0.1 U/kg/h) + restricted fluids (<1500 mL/day); monitoring: hourly urine output, every 2 h sodium, continuous arterial pressure. (2) Grade II (moderate DI, hemodynamically stable): criteria: urine output 4 – 6 L/day + MAP ≥65 mmHg; intervention: Pituitrin bolus (0.05 U/kg every 6 h) + ad libitum oral intake; monitoring: every 4 h urine output, daily sodium. (3) Grade III (Mild/Thoracic SCI above T5 DI): criteria: urine output <4 L/day or thoracic SCI; intervention: desmopressin (1 – 2 μg IV every 12 h); monitoring: every 8 h urine specific gravity.

### 4.7 Strengthened limitations & future directions

This retrospective study has inherent limitations. Selection bias, heterogeneous control group management (mixed desmopressin/no-treatment), and limited subgroup power constrain generalizability. Future multicenter randomized controlled trials with standardized protocols and expanded cohorts are required to validate findings. We propose developing machine learning-based dosing models (integrating ASIA grade, injury level, and hemodynamics) through prospective registries (registration pending), enabling precision delivery of dual-receptor therapy in SCI-related DI. As this was a single-center retrospective pilot study with a relatively small matched cohort, the results should be interpreted with caution. Further studies are necessary to confirm these findings and establish standardized protocols.

This study focused primarily on short-term outcomes, including DI resolution, mortality, and hospitalization. While early hemodynamic stabilization theoretically contributes to spinal cord perfusion and secondary injury mitigation, ASIA grade evolution was not longitudinally tracked beyond discharge in this cohort. Future trials should incorporate long-term neurological follow-up to assess functional recovery and validate potential neuroprotective benefits of Pituitrin.

In conclusion, this study provides mechanistic evidence that early Pituitrin administration reduces DI duration, mortality, and hospitalization in acute SCI through dual V1a/V2 receptor activation—a strategic advance over conventional therapies. Our stratified protocol (Grade I – III) and predictive dosing framework advance precision management of SCI-induced neuroendocrine crises. These preliminary findings suggest that Pituitrin may offer a promising therapeutic approach for addressing the hemodynamic-endocrine interplay in acute SCI, although validation through larger, prospective multicenter trials is needed.

## CRediT authorship contribution statement

**Lianhua Li:** Writing – original draft, Methodology, Investigation, Conceptualization. **Jie Gao:** Formal analysis, Data curation. **Hao Wang:** Visualization, Methodology. **Zhonghe Wang:** Methodology, Formal analysis, Data curation. **Hua Chen:** Resources, Project administration. **Zhi Liu:** Writing – review & editing.

## Ethical statement

This study was performed in accordance with the ethical standards of the institutional and national research committee and with the 1964 Helsinki Declaration and its later amendments or comparable ethical standards. The study was reviewed and approved by the institutional ethics board of Chinese People's Liberation Army (PLA) General Hospital. All authors certify that the methods were carried out in accordance with relevant guidelines and regulations. All authors certify that the study was reviewed and approved by the hospital.

## Funding

This study had no financial support.

## Declaration of competing interest

The authors have no relevant financial or non-financial interests to disclose. The authors have no competing interest to declare that is relevant to the content of this article. All authors certify that they have no affiliations with or involvement in any organization or entity with any financial interest or non-financial interest in the subject matter or materials discussed in this manuscript. The authors have no financial or proprietary interests in any material discussed in this article.
